# Supplementary material for: Determination of Genetically Identical Strains of Four Honeybee Viruses in Bumblebee Positive Samples
Source: Viruses. 2020 Nov 16;12(11):1310. doi: 10.3390/v12111310 (PMC7697897; doi:10.3390/v12111310)
Supplement: Supplementary file 1 [file viruses-12-01310-s001.pdf]

Determination of genetically identical strains of four honeybee viruses in bumblebee positive samples

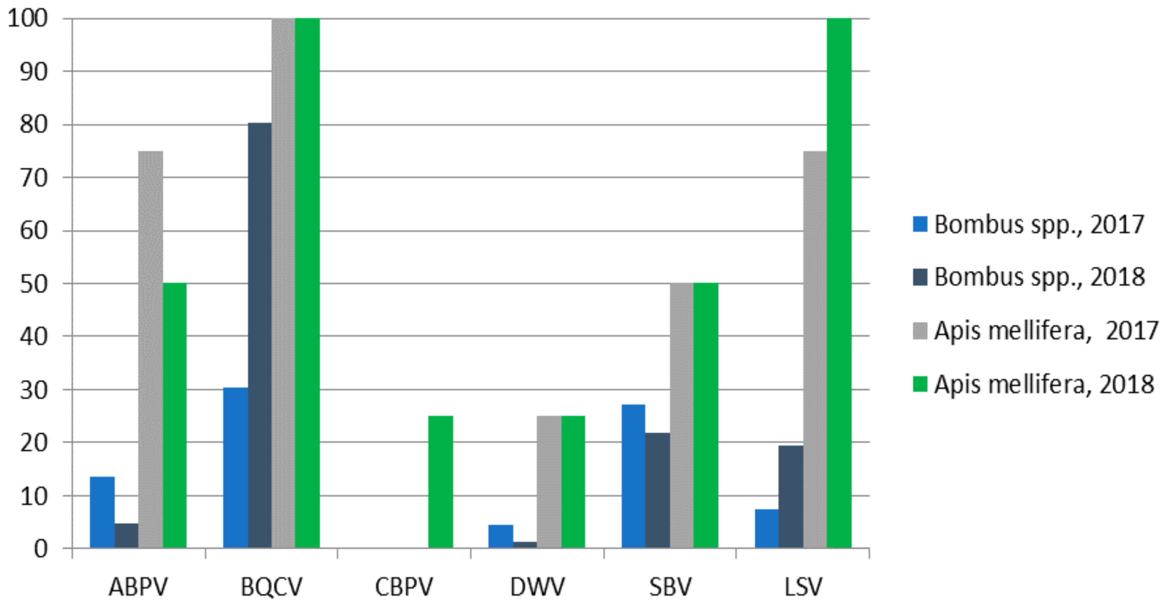

Figure S1: The percentages of detected positive samples for ABPV-, BQCV-, CBPV-, DWV-, SBV-, and LSV-specific RT-PCR methods presented for *Bombus* spp. and *Apis mellifera* collected in August 2017 and August 2018.

Table S1: 33 sequences of ABPV from Slovenia in GenBank

ABPV	Host	Name of sample	Date of sample collection	Archive /Location	GenBank accession number
Seq1	*Apis mellifera carnica*	246/2016	7 October 2016	Monitoring	MH900021
Seq2	*Apis mellifera carnica*	247/2016	17 October 2016	Monitoring	MH900022
Seq3	*Apis mellifera carnica*	248/2016	14 October 2016	Monitoring	MH900023
Seq4	*Apis mellifera carnica*	249/2016	14 October 2016	Monitoring	MH900024
Seq5	*Apis mellifera carnica*	250/2016	19 October 2016	Monitoring	MH900025
Seq6	*Apis mellifera carnica*	251/2016	26 October 2016	Monitoring	MH900026
Seq7	*Apis mellifera carnica*	252/2016	26 October 2016	Monitoring	MH900027
Seq8	*Apis mellifera carnica*	253/2016	2 October 2016	Monitoring	MH900028
Seq9	*Apis mellifera carnica*	254/2016	2 October 2016	Monitoring	MH900029
Seq10	*Apis mellifera carnica*	255/2016	26 October 2016	Monitoring	MH900030
Seq11	*Apis mellifera carnica*	256/2016	4 November 2016	Monitoring	MH900031
Seq12	*Apis mellifera carnica*	257/2016	10 November 2016	Monitoring	MH900032
Seq13	*Apis mellifera carnica*	264/2016	21 November 2016	Monitoring	MH900033
Seq14	*Apis mellifera carnica*	267/2016	23 November 2016	Monitoring	MH900034
Seq15	*Apis mellifera carnica*	268/2016	5 December 2016	Monitoring	MH900035
Seq16	*Apis mellifera carnica*	269/2016	7 December 2016	Monitoring	MH900036
Seq17	*Apis mellifera carnica*	270/2017	3 January 2017	Monitoring	MH900037
Seq18	*Apis mellifera carnica*	271/2017	13 January 2017	Monitoring	MH900038
Seq19	*Apis mellifera carnica*	274/2017	17 February 2017	Monitoring	MH900039
Seq20	*Apis mellifera carnica*	275/2017	17 February 2017	Monitoring	MH900040
Seq21	*Apis mellifera carnica*	276/2017	24 February 2017	Monitoring	MH900041
Seq22	*Apis mellifera carnica*	277/2017	6 March 2017	Monitoring	MH900042
Seq23	*Apis mellifera carnica*	278/2017	23 March 2017	Monitoring	MH900043
Seq24	*Apis mellifera carnica*	281/2017	10 May 2017	Monitoring	MH900044
Seq25	*Apis mellifera carnica*	284/2017	30 July 2017	Monitoring	MH900045
Seq26	*Bombus spp.*	Bombus-287/2017	15 July 2017	Monitoring	MH900046
Seq27	*Bombus spp.*	Bombus-289/2017	15 July 2017	Monitoring	MH900047
Seq28	*Bombus spp.*	Bombus-290/2017	15 July 2017	Monitoring	MH900048
Seq29	*Bombus lapidarius*	Bombus-BL3/2017	09 August 2017	Location 1	MH900049
Seq30	*Bombus lapidarius*	Bombus-BL4/2017	09 August 2017	Location 1	MH900050
Seq31	*Bombus pascuorum*	Bombus-BP4/2017	09 August 2017	Location 1	MH900051
Seq32	*Bombus terrestris*	Bombus-BT23/2017	23 August 2017	Location 3	MH900052
Seq33	*Bombus terrestris*	Bombus-BT29/2017	23 August 2017	Location 3	MH900053

Table S2: 75 sequences of BQCV from Slovenia in GenBank

BQCV	Host	Name of sample	Date of sample collection	Archive /Location	GenBank accession number
Seq1	*Apis mellifera carnica*	1/2008	12 October 2008	Monitoring	MH899946
Seq2	*Apis mellifera carnica*	2/2009	25 April 2009	Monitoring	MH899947
Seq3	*Apis mellifera carnica*	3/2009	4 May 2009	Monitoring	MH899948
Seq4	*Apis mellifera carnica*	4/2009	4 May 2009	Monitoring	MH899949
Seq5	*Apis mellifera carnica*	5/2009	22 May 2009	Monitoring	MH899950
Seq6	*Apis mellifera carnica*	6/2009	29 May 2009	Monitoring	MH899951
Seq7	*Apis mellifera carnica*	115/2009	2 June 2009	Monitoring	MH899952
Seq8	*Apis mellifera carnica*	116/2009	2 June 2009	Monitoring	MH899953
Seq9	*Apis mellifera carnica*	129/2008	20 January 2008	Monitoring	MH899954
Seq10	*Apis mellifera carnica*	153/2009	13 June 2009	Monitoring	MH899955
Seq11	*Apis mellifera carnica*	163-1/2008	24 May 2008	Monitoring	MH899956
Seq12	*Apis mellifera carnica*	163-2/2008	24 May 2008	Monitoring	MH899957
Seq13	*Apis mellifera carnica*	164/2008	24 May 2008	Monitoring	MH899958
Seq14	*Apis mellifera carnica*	165/2008	24 May 2008	Monitoring	MH899959
Seq15	*Apis mellifera carnica*	166/2008	24 May 2008	Monitoring	MH899960
Seq16	*Apis mellifera carnica*	172/2007	17 Marchj 2007	Monitoring	MH899961
Seq17	*Apis mellifera carnica*	246/2016	7 October 2016	Monitoring	MH899962
Seq18	*Apis mellifera carnica*	247/2016	17 October 2016	Monitoring	MH899963
Seq19	*Apis mellifera carnica*	248/2016	14 October 2016	Monitoring	MH899964
Seq20	*Apis mellifera carnica*	249/2016	14 October 2016	Monitoring	MH899965
Seq21	*Apis mellifera carnica*	253/2016	2 November 2016	Monitoring	MH899966
Seq22	*Apis mellifera carnica*	254/2016	2 November 2016	Monitoring	MH899967
Seq23	*Apis mellifera carnica*	257/2016	10 November 2016	Monitoring	MH899968
Seq24	*Apis mellifera carnica*	258/2016	10 November 2016	Monitoring	MH899969
Seq25	*Apis mellifera carnica*	264/2016	21 November 2016	Monitoring	MH899970
Seq26	*Apis mellifera carnica*	267/2016	23 November 2016	Monitoring	MH899971
Seq27	*Apis mellifera carnica*	269/2016	7 December 2016	Monitoring	MH899972
Seq28	*Apis mellifera carnica*	274/2017	17 February 2017	Monitoring	MH899973
Seq29	*Apis mellifera carnica*	275/2017	17 February 2017	Monitoring	MH899974
Seq30	*Apis mellifera carnica*	276/2017	24 February 2017	Monitoring	MH899975
Seq31	*Apis mellifera carnica*	278/2017	23 March 2017	Monitoring	MH899976
Seq32	*Apis mellifera carnica*	279/2017	21 April 2017	Monitoring	MH899977
Seq33	*Apis mellifera carnica*	280/2017	5 May 2017	Monitoring	MH899978
Seq34	*Apis mellifera carnica*	281/2017	10 May 2017	Monitoring	MH899979
Seq35	*Apis mellifera carnica*	284/2017	30 July 2017	Monitoring	MH899980
Seq36	*Apis mellifera carnica*	285/2007	13 June 2007	Monitoring	MH899981
Seq37	*Apis mellifera carnica*	286/2007	14 June 2007	Monitoring	MH899982
Seq38	*Apis mellifera carnica*	287-1/2007	14 June 2007	Monitoring	MH899983
Seq39	*Apis mellifera carnica*	287-2/2007	14 June 2007	Monitoring	MH899984
Seq40	*Apis mellifera carnica*	287-3/2007	14 June 2007	Monitoring	MH899985
Seq41	*Apis mellifera carnica*	287/2017	15 July 2017	Monitoring	MH899986
Seq42	*Apis mellifera carnica*	292/2017	28 September 2017	Monitoring	MH899987
Seq43	*Apis mellifera carnica*	296/2017	4 December 2017	Monitoring	MH899988
Seq44	*Apis mellifera carnica*	297/2018	5 January 2018	Monitoring	MH899989
Seq45	*Apis mellifera carnica*	301/2018	3 January 2018	Monitoring	MH899990
Seq46	*Apis mellifera carnica*	0409/2009	4 September 2009	Monitoring	MH899991
Seq47	*Apis mellifera carnica*	0610-2/2009	6 October 2009	Monitoring	MH899992
Seq48	*Apis mellifera carnica*	0610-4/2009	6 October 2009	Monitoring	MH899993
Seq49	*Apis mellifera carnica*	637/2009	22 May 2009	Monitoring	MH899994
Seq50	*Apis mellifera carnica*	1930-1/2009	12 August 2009	Monitoring	MH899995

Seq51	*Apis mellifera carnica*	1956-1/2009	18 August 2009	Monitoring	MH899996
Seq52	*Apis mellifera carnica*	1957-1/2009	18 August 2009	Monitoring	MH899997
Seq53	*Apis mellifera carnica*	1960-1/2009	26 August 2009	Monitoring	MH899998

BQCV	Host	Name of sample	Date of sample collection	Archive /Location	GenBank accession number
Seq54	*Apis mellifera carnica*	1960-4/2009	26 August 2009	Monitoring	MH899999
Seq55	*Apis mellifera carnica*	1960-11/2009	26 August 2009	Monitoring	MH900000
Seq56	*Apis mellifera carnica*	01234-4/2009	29 August 2009	Monitoring	MH900001
Seq57	*Apis mellifera carnica*	01234-6/2009	29 August 2009	Monitoring	MH900002
Seq58	*Apis mellifera carnica*	GRM/2017	9 August 2017	Location 1	MH900003
Seq59	*Apis mellifera carnica*	LJU/2017	28 August 2017	Location 4	MH900004
Seq60	*Apis mellifera carnica*	LUK/2017	10 August 2017	Location 2	MH900005
Seq61	*Apis mellifera carnica*	NAK/2017	10 August 2017	Location 3	MH900006
Seq62	*Bombus spp.*	Bombus-290/2017	15 July 2017	Monitoring	MH900007
Seq63	*Bombus lapidarius*	Bombus-BL2/2017	9 August 2017	Location 1	MH900008
Seq64	*Bombus lapidarius*	Bombus-BL4/2017	9 August 2017	Location 1	MH900009
Seq65	*Bombus lapidarius*	Bombus-BL6/2017	9 August 2017	Location 1	MH900010
Seq66	*Bombus lapidarius*	Bombus-BL7/2017	9 August 2017	Location 1	MH900011
Seq67	*Bombus pascuorum*	Bombus-BP22/2017	28 August 2017	Location 4	MH900012
Seq68	*Bombus pascuorum*	Bombus-BP23/2017	28 August 2017	Location 4	MH900013
Seq69	*Bombus terrestris*	Bombus-BT23/2017	22 August 2017	Location 3	MH900014
Seq70	*Bombus terrestris*	Bombus-BT27/2017	22 August 2017	Location 3	MH900015
Seq71	*Bombus terrestris*	Bombus-BT29/2017	22 August 2017	Location 3	MH900016
Seq72	*Bombus terrestris*	Bombus-BT30/2017	22 August 2017	Location 3	MH900017
Seq73	*Bombus terrestris*	Bombus-BT33/2017	22 August 2017	Location 4	MH900018
Seq74	*Apis mellifera carnica*	SUK/2009	9 December 2009	Monitoring	MH900019
Seq75	*Apis mellifera carnica*	VLA-4/2009	18 December 2009	Monitoring	MH900020

Table S3: 25 sequences of SBV from Slovenia in GenBank

SBV	Host	Name of sample	Date of sample collection	Archive /Location	GenBank accession number
Seq1	*Apis mellifera carnica*	115/2009	2 June 2009	Monitoring	MH900054
Seq2	*Apis mellifera carnica*	116/2009	2 June 2009	Monitoring	MH900055
Seq3	*Apis mellifera carnica*	153/2009	13 June 2009	Monitoring	MH900056
Seq4	*Apis mellifera carnica*	289/2007	14 June 2007	Monitoring	MH900057
Seq5	*Apis mellifera carnica*	AZ1/2016	14 June 2016	Monitoring	MH900058
Seq6	*Apis mellifera carnica*	AZ2/2016	14 June 2016	Monitoring	MH900059
Seq7	*Apis mellifera carnica*	LJU/2017	28 August 2017	Location 4	MH900060
Seq8	*Apis mellifera carnica*	N1/2016	14 June 2016	Monitoring	MH900061
Seq9	*Apis mellifera carnica*	N2/2016	14 June 2016	Monitoring	MH900062
Seq10	*Apis mellifera carnica*	N5/2016	14 June 2016	Monitoring	MH900063
Seq11	*Apis mellifera carnica*	NAK/2017	10 August 2017	Location 3	MH900064
Seq12	*Bombus pascuorum*	Bombus-BP11/2017	10 August 2017	Location 3	MH900065
Seq13	*Bombus pascuorum*	Bombus-BP12/2017	10 August 2017	Location 3	MH900066
Seq14	*Bombus pascuorum*	Bombus-BP14/2017	10 August 2017	Location 3	MH900067
Seq15	*Bombus pascuorum*	Bombus-BP16/2017	10 August 2017	Location 3	MH900068
Seq16	*Bombus pascuorum*	Bombus-BP17/2017	10 August 2017	Location 3	MH900069
Seq17	*Bombus pascuorum*	Bombus-BP18/2017	10 August 2017	Location 3	MH900070
Seq18	*Bombus pascuorum*	Bombus-BP19/2017	10 August 2017	Location 3	MH900071
Seq19	*Bombus pascuorum*	Bombus-BP20/2017	10 August 2017	Location 3	MH900072
Seq20	*Bombus terrestris*	Bombus-BT15/2017	23 August 2017	Location 2	MH900073
Seq21	*Bombus terrestris*	Bombus-BT23/2017	22 August 2017	Location 3	MH900074
Seq22	*Bombus terrestris*	Bombus-BT24/2017	22 August 2017	Location 3	MH900075
Seq23	*Bombus terrestris*	Bombus-BT27/2017	24 August 2017	Location 3	MH900076
Seq24	*Bombus terrestris*	Bombus-BT28/2017	24 August 2017	Location 3	MH900077
Seq25	*Bombus terrestris*	Bombus-BT33/2017	28 August 2017	Location 4	MH900078

Table S4: 25 sequences of LSV from Slovenia in GenBank

LSV	Host	Name of sample	Date of sample collection	Archive /Location	GenBank accession number
Seq1	*Apis mellifera*	LSV3/CB281/2017	10 May 2017	Monitoring	MH350870
Seq2	*Bombus terrestris*	LSV3/BombusBT35/2017	28 August 2017	Location 4	MH350871
Seq3	*Apis mellifera*	LSV3/CB274/2017	17 February 2017	Monitoring	MH350872
Seq4	*Apis mellifera*	LSV3/CB280/2017	5 May 2017	Monitoring	MH350873
Seq5	*Apis mellifera*	LSV3/CB270/2017	3 January 2017	Monitoring	MH350874
Seq6	*Apis mellifera*	LSV3/CB264/2016	21 November 2016	Monitoring	MH350875
Seq7	*Apis mellifera*	LSV3/CB276/2017	24 February 2017	Monitoring	MH350876
Seq8	*Apis mellifera*	LSV3/CB252/2016	26 October 2016	Monitoring	MH350877
Seq9	*Apis mellifera*	LSV3/CBLJ/2017	28 August 2017	Location 4	MH350878
Seq10	*Apis mellifera*	LSV3/CB249/2016	14 October 2016	Monitoring	MH350879
Seq11	*Apis mellifera*	LSV3/CB284/2017	30 July 2017	Monitoring	MH350880
Seq12	*Apis mellifera*	LSV3/CB271/2017	13 January 2017	Monitoring	MH350881
Seq13	*Apis mellifera*	LSV3/CBNA/2017	10 August 2017	Location 3	MH350882
Seq14	*Bombus pascuorum*	LSV3/BombusBP9/2017	10 August 2017	Location 2	MH350883
Seq15	*Apis mellifera*	LSV1/CB272/2017	14 February 2017	Monitoring	MH350884
Seq16	*Apis mellifera*	LSV2/CB247/2016	17 October 2016	Monitoring	MH350885
Seq17	*Apis mellifera*	LSV2/CB257/2016	10 November 2016	Monitoring	MH350886
Seq18	*Apis mellifera*	LSV2/CB269/2016	7 December 2016	Monitoring	MH350887
Seq19	*Apis mellifera*	LSV2/CB277/2017	6 March 2017	Monitoring	MH350888
Seq20	*Bombus terrestris*	LSV2/BombusBT21/2017	22 August 2017	Location 3	MH350889
Seq21	*Apis mellifera*	LSV2/CB286/2017	4 September 2017	Monitoring	MH350890
Seq22	*Apis mellifera*	LSV2/CB300/2018	9 January 2018	Monitoring	MH350891
Seq23	*Apis mellifera*	LSV2/CB279/2017	2 April 2017	Monitoring	MH350892
Seq24	*Apis mellifera*	LSV2/CB278/2017	23 March 2017	Monitoring	MH350893
Seq25	*Apis mellifera*	LSV2/CB250/2016	19 October 2016	Monitoring	MH350894

Table S5: The percentage of detected positive samples for ABPV, BQCV, CBPV, DWV, SBV, and LSV by specific RT-PCR method for *Bombus spp.* collected in August 2017 and 2018 at four different geographic areas (Location 1 - 4) in Slovenia.

Location	Species	No of samples	Year	**ABPV**	**BQCV**	**CBPV**	**DWV**	**SBV**	**LSV**
Location 1	*Bombus spp.*	24	2017	25%	25%	0%	12,50%	0%	0%
Location 2	*Bombus spp.*	11	2017	9%	0%	0%	0,00%	9%	0%
Location 3	*Bombus spp.*	20	2017	10%	40%	0%	0,00%	80%	10%
Location 4	*Bombus spp.*	11	2017	0%	55%	0%	0,00%	9%	18%
**Total**		**n=66**	**2017**	**13.64%**	**30.30%**	**0%**	**4.55%**	**27.27%**	**7.32%**
Location 1	*Bombus spp.*	30	2018	0%	87%	0%	0,00%	0%	13%
Location 2	*Bombus spp.*	15	2018	0%	73%	0%	0,00%	0%	47%
Location 3	*Bombus spp.*	17	2018	11,8%	82,4%	0,0%	5,9%	58,8%	11,8%
Location 4	*Bombus spp.*	20	2018	10,0%	80,0%	0,0%	0,0%	40,0%	20,0%
**Total**		**n=82**	**2018**	**4.88%**	**80.49%**	**0%**	**1.22**	**21.95%**	**19.51%**
